# A Life Course Model of Self-Reported Violence Exposure and Ill-health with A Public Health Problem Perspective

**DOI:** 10.3934/publichealth.2014.1.9

**Published:** 2014-01-27

**Authors:** Niclas Olofsson

**Affiliations:** Department of Research and Development, Härnösand, 871 85 Härnösand, Sweden. E-mail: niclas.olofsson@lvn.se; Tel: +46-611-80-078

**Keywords:** self-reported violence, life course, ill-health, accumulation, transition, stress

## Abstract

Violence has probably always been part of the human experience. Its impact can be seen, in various forms, in all parts of the world. In 1996, WHO:s Forty-Ninth World Health Assembly adopted a resolution, declaring violence a major and growing public health problem around the world. Public health work centers around health promotion and disease prevention activities in the population and public health is an expression of the health status of the population taking into account both the level and the distribution of health. Exposure to violence can have many aspects, differing throughout the life course ― deprivation of autonomy, financial exploitation, psychological and physical neglect or abuse — but all types share common characteristics: the use of destructive force to control others by depriving them of safety, freedom, health and, in too many instances, life; the epidemic proportions of the problem, particularly among vulnerable groups; a devastating impact on individuals, families, neighborhoods, communities, and society. There is considerable evidence that stressful early life events influence a variety of physical and/or psychological health problems later in life. Childhood adversity has been linked to elevated rates of morbidity and mortality from number of chronic diseases. A model outlining potential biobehavioural pathways is put forward that may be a potential explanation of how exposure to violence among both men and women work as an important risk factor for ill health and should receive greater attention in public health work.

## 1. Introduction

Every year, more than 1.6 million people worldwide lose their lives to violence. For every person who dies as a result of violence, many more are injured and suffer from a range of physical, sexual, reproductive and mental health problems [1]. Violence places a massive burden on national economies, individuals, families, communities and society, costing countries billions of US dollars each year in health care, law enforcement and lost productivity [2],[3],[4],[5]. In the United States alone, estimates of the costs of violence reach 3.3% of the GDP (estimated GDP 2005; 12.4 trillion US dollars) [3].

Despite the fact that violence has always been present and is among the leading causes of death worldwide for people aged 15–44, violence have been neglected from the global health agenda for many years, although being predictable and largely preventable [6]. However, as long as there has been violence, there have also been societal systems ― religious, philosophical, legal and communal ― that have grown to prevent or limit it. None has been completely successful, but all have made their contributions. Since the early 1980s, the field of public health has been a growing asset in this response to battle violence. The focus of public health is on the safety and well-being of entire populations. A unique aspect of the field is that it strives to provide services that benefit the largest number of people [7]. Violence can be prevented and its impact reduced, in the same way that public health efforts have prevented and reduced pregnancy-related complications, workplace injuries, infectious diseases and illness resulting from contaminated food and water in many parts of the world [1]. The public health approach is a four-step process that is well rooted in the scientific method. The first step in preventing violence is to understand the “who”, “what”, “when”, “where” and “how” associated with it. It is important, as a second step to understand what factors protect people or put them at risk for experiencing or perpetrating violence. In a third step prevention programs must be developed as well as tested. Once the prevention programs have been proven effective in a fourth step, they must be implemented and adopted more broadly in the communities [1],[7].

Each individual life is unique, but everyone goes through the same basic sequence. The course of life have qualitatively different life-stages with a beginning, middle and an end [8],[9]. The perspective of a life cycle tries to relate the place where the individual is in the course of his or her life with the kind of issues they are facing and the individual resources available to them to help them face these issues, as well as the possible disturbance that might develop if they fail to cope successfully with the issues [10],[11]. The life-course perspective focuses on understanding how early-life experiences can shape health across an entire lifetime and potentially across generations; it systematically directs attention to the role of context, including social and physical context along with biological factors, over time [12]. That is, the life course perspective is an example of a developmental perspective that can be used to conceptualize processes through which earlier life experiences influence later health [13],[14].

Exposure to violence can have many consequences, differing throughout the life course — deprivation of autonomy, financial exploitation, psychological and physical neglect or abuse — but all types share common characteristics: 1) the use of destructive force to control others by depriving them of safety, freedom, health and, in too many instances, life; 2) the epidemic proportions of the problem, particularly among vulnerable groups; 3) the potential for intergenerational transmission; and 4) a devastating impact on individuals, families, neighbourhoods, communities and society[15],[16],[17],[18],[19],[20].

The consequences of child abuse, violence exposure during adolescence or young adulthood, intimate partner violence and elderly abuse are commonly encountered within the health care system [21],[22],[23],[24],[25],[26]. In the past, these different types of violence exposure have been studied in isolation. More recently it has become apparent that they are often closely interconnected [27]. Interventions directed at one form of violence may be beneficial to others as well [21],[22],[28]. Being born into a social and physical hazardous environment in Bangladesh in 2000 is likely to be associated with very different early life exposures than being born into social and physical hazardous environment in the United States in the 1950s. The social meaning and the means to deal with physical hazards, in connection to its life course links to particular types of exposures, as well as the prevailing disease environment will all influence the potential for early life factors to be expressed in different adverse outcomes later in life.

The understanding and “who”, “what”, “when”, “where” and “how” concerning violence and ill-health are dealt with by a public health approach while the conceptualization of the developmental aspects of “who”, “what”, “when”, “where” and “how” are put in a life course perspective.

The general aim of this mini-review is to briefly summarize work demonstrating that the life-course perspective could facilitate the understanding of the detrimental relation between exposure to violence or threats of violence and ill health at different ages and in different time periods of life. Throughout this mini-review violence is generally defined as any type of self-reported violence or threats of violence [1]. Specifically the mini-review focus on the relation between self-reported violence and ill-health through four life-stages; childhood and adolescence, young adulthood, adulthood, and elderly. Ill health is, in this mini-review, defined as self-reported health or expressed as self-reported physical and psychological symptoms. A model will be proposed were different bio behavioural pathways suggests giving one potential explanation of self-reported violence exposure through life course and the relation to adult ill-health. Finally, ideas for future work to test different aspects of the model will be suggested.

## 2. Understanding the association between self-reported exposure to violence and ill health

In trying to understand early adversity (specifically violence and threats of violence), two earlier findings need to be taken into account, since they have demonstrated 1) that many children living in a family where the mother is exposed to domestic violence are frequently abused themselves and 2) that violence-exposed women are often insufficient caregivers [17],[29], which could affect the children regardless of whether they have seen the violent act or not. Violence against women may also have indirect negative effects on their children. Women exposed to violence or threats experience physical and mental health impacts and depression [18],[30],[31]. Maternal depression may also have negative health effects on children, including increased illness [32], increases in health care utilization [33],[34], poorer health status [35], and greater risk of mental health problems [36],[37]. Furthermore, associations between childhood maltreatment and post-traumatic stress and emotional distress in the children have been described [38]. Several authors have pointed out that these children are in fact often exposed to several other stressors, such as negative disclosures about the family or economic and social disadvantages [17].

Subsequent research has suggested that post-traumatic stress is a plausible biological mechanism for negative physical health outcomes and that post-traumatic stress symptoms tend to take on a mediating role or add negative physical and psychological effects to the children [39],[40],[41],[42],[43],[44],[45],[46].

Linton and associates have in several articles discussed the association between exposure to violence or threats of violence and the experience of pain and ability to cope with pain [47],[48],[49]. Other researchers have proposed that ill health in connection to self-reported exposure to violence could be due to increased somatization [50]. In an article in The Lancet, Campbell discusses the impact of increased stress: strained psychological health might influence the immune system which in turn might affect the persońs health in a negative way [17],[51]. Several earlier studies have highlighted the negative association between a heightened level of stress and the immune system and health [52],[53],[54],[55].

Concerning young adults, other possible inputs to understand and other possible associations between self-reported exposure to violence and ill health have been presented by research. The vast majority of the men exposed to violence, in a study by Olofsson et. al. (2009), had been subjected in a public place, meaning that this violence was probably not inflicted by an intimate partner, while 40% of the women exposed to violence had been exposed in a domestic setting and a higher percentage may thus have been the victims of violence inflicted by an intimate partner. This results taken together with other research exploring gender differences substance abuse and violence [56],[57],[58], indicates that violence against young women often differs from that against young men, frequently occurring at other places and possibly in other situations, with consequences that are more serious for the health of the women. Young men and women do not face equal risks of exposure to violence. There were significant differences for all socioeconomic variables and the use of various drugs for those exposed to violence compared with those who were not. The violence-exposed young men had more often hazardous drinking patterns [59]. It was impossible though, to tell from that study whether the violence was experienced in connection with drinking or whether the alcohol was used, for example, to reduce pain or anxiety after an experience of violence or threats, as discussed by Campbell (2002), in which it is pointed out that physical abuse may contribute to both cigarette and substance abuse [17],[51].

When analysing an elderly population, physical abuse was more strongly associated with men's self-reported ill health. This could have reflected an actual lower association among women between physical abuse and self-reported health, but could as likely have been an artefact caused by the low prevalence of physical abuse among women [60]. The demonstrated strong association between psychological abuse and self-rated health in that study resembled the results in another study [61]. Indeed, being psychologically abused seems to be a stronger negative predictor of poor self-rated health, when comparing to being exposed to physical violence [62],[63],[64],[65]. Psychological symptoms such as anxiety and depressive symptoms have also been shown to significantly mediate the effect on health status [66],[67],[68].

Although perceiving fear of crime seems to have little connection to victimization [60],[69],[70],[71], the actual perception and fear of being exposed to a crime strongly relate to ill health both in women and men. This might suggest that the experience of fear of crime could lead to poor health through psychosocial mechanisms like stress and that the mechanisms are shared by both women and men [72].

## 3. Exposure to violence and life course health

An expanded way of possibly understanding a life-course view of the relation between self-reported exposure to violence and ill-health is needed [71]. Although an association between socioeconomic condition, social disadvantages, and other stressful life events with health problems has been demonstrated elsewhere [15],[18],[73],[74],[75],[76],[77],[78],[79],[80],[81],[82],[83],[84],[85],[86],[87],[88],[89],[90],[91],[92],[93],[94],[95], the underlying causal mechanisms have remained unclear. There are arrays of mechanisms through which experiences of child abuse or violence in adolescence, for instance, can jeopardize individuals' functioning well into adulthood [96],[97]. Focusing specifically on adult physical health, there are four trajectories through which early exposure to violence can lead to poorer adult physical health, namely, behavioural trajectories (e.g., excessive drinking, substance abuse, or smoking), social trajectories (e.g., homelessness and repeated victimizations), cognitive pathways (e.g., troubled early attachment, learning difficulties, externalizing or internalizing problems), and emotional trajectories (e.g., depressive symptoms or post-traumatic stress symptoms (PTSD) [96],[98]. An ecobiodevelopmental model proposed by Garner and Shonkoff [99],[100] illustrates how early experiences and environmental influences could leave lasting signatures on the genetic predispositions that affect the developing brain and the future health. In relation to accumulating traumatic childhood or adolescent events, family characteristics (such as parental psychopathology, parental loss or absence, or parental divorce) during childhood could contribute to the development of subsequent future health-related well-being or problems in adulthood [101],[102]. Also, persons who have experienced adversities during their upbringing are more likely to participate in high-risk behaviours [96],[102], which are related to both ill health and violence exposure [103]. Miller et. al. (2011) have presented a ‘Biological Embedding Model’ which synthesized knowledge to be able to address the question ― why do early psychological stressors co-vary with elevated rates of morbidity and mortality from chronic disease of aging [104].

Continual psychological pressure and/or persistent wear and tear of the body due to repeated stressful or traumatic experiences over the life course might dysregulate the normal physiological adaptations to stress and threats, and later sensitivity to stress [105],[106],[107],[108],[109], or influence immune functioning which may in turn contribute to increased adult health problems [107],[110].

## 4. Understanding the complex association between self-reported exposure to violence and ill health: summing up with a model

**Figure 1.**
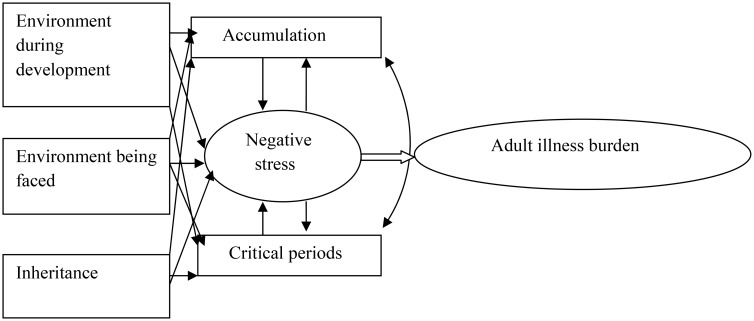
A possible model of understanding of the association between violence exposure and ill health

The life-course perspective generally refers to the interweave of age-graded trajectories, such as work careers and family pathways, that are subject to changing conditions and future options, and to short-term transitions ranging from leaving school to retirement [95]. Trying to fit together all parts and connections of the life course into a model would be tremendously difficult. However, different main parts with interconnections could be fitted into a model, where they could be potentially confirmed and supported with references. Research supports the different main parts of the model in Figure 1; one's upbringing during childhood/adolescence lays the foundation in the *environment during development* [99],[111],[112] and its association to different life course pathways (accumulating negative experiences such as exposure to violence or positive experiences during potentially critical periods) and negative stress [78],[99],[113],[114]. But, it is not only upbringing that shapes the life course. There are a present *environment being faced* [82],[99],[111],[112],[115],[116],[117] and a past including an *inheritance* [76],[118],[119]. Different exposures in different time periods are intertwined in an *accumulating* fashion [28],[83],[85],[99],[112],[120] or in certain *critical periods* [74],[75],[96],[99],[106],[121],[122] with potentially *negative stress* as outcomes [101],[105],[107],[123],[124],[125],[126],[127],[128]. Eventual wear and tear over the life course might end up in *adult illness burden* [14],[97],[109],[110],[129].

**Figure 2.**
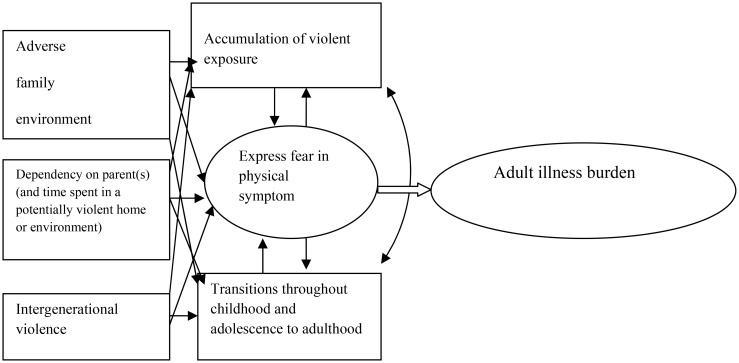
Trying to fit empirical research on violence exposure and ill health into the theoretical model

In Figure 2 this complex network is exemplified through different empirical results in another model. Children living in a home where they are exposed to family violence have a higher risk of ending up in a violent relationship [104],[130]. As children spend most of their time at home in younger years, it is a possible that this causes them to be exposed to the adverse family environments more often [41],[104]. A potentially higher health risk in younger children is that the younger the child, the more dependent it is on the mother and/or father for its well-being [131]. Age influences the way children make sense of their experiences and at a younger age, children are more likely to express their fears in physical symptoms [132],[133]. As the number of adverse violent experiences cumulate over time, a graded relationship between PTSD, chronic medical conditions and the risk of severe adult illness burden increases [104],[129]. It seems that even type of abuse exposures are linked to specific effects during several steps of transitions and critical periods [122]. During these critical times of victimization in childhood and adolescence, several other areas of age-related activities suffer, such as educational performance [104],[134].

**Table 1.** Comparing self-reported health outcomes throughout the life cycle with reported exposure to violence verses non-exposure of violence, adjusted OR, and 95% CI. Significant raised OR in bold print

	Children	Young adults	Adults	Adults	Elderly
	0-18 y^1^	18-29y^2^	30-44y^3^	45-64y^5^	65-84y^4^
	Girls/Boys	Women/Men	Women/Men	Women/Men	Women/Men
Stomach ache	**2.2 (1.5-3.2) 1.7 (1.1-2.7)**	**1.3 (1.3-1.4)**	**6.6 (5.4-7.9)**	**1.4 (1.1-1.7**	**1.5 (1.0-2.4)**
		**0.5 (0.5-0.6)**	**1.2 (0.9-1.6)**	**1.3 (0.9-1.7)**	**1.0 (0.6-1.7)**
Diffuse muscular pain	**2.2 (1.7-2.9)**	**3.1 (3.0-3.3)**	0.8 (0.8-0.9)	**1.4 (1.1-1.7)**	1.1 (0.7-1.9)
	**1.4 (1.0-1.8)**	**3.8 (3.4-4.3)**	**1.6 (1.4-1.7)**	**1.8 (1.4-2.4)**	**2.6 (1.8-4.1)**
Allergy/asthma	**1.7 (1.1-2.7)**	**2.3 (2.1-2.5)**	N.A.	**1.4 (1.1-1.7)**	**1.9 (1.1-3.2)**
	0.8 (0.4-1.7)	0.9 (0.8-1.1)	**7.9 (6.8-9.2)**	1.2 (0.9-1.7)	1.2 (0.7-2.3)
Anxiety	**6.1 (5.1-7.2)**	**1.6 (1.5-1.6)**	**2.9 (2.6-3.3)**	**2.6 (1.8-3.7) 3.0 (1.9-4.8)**	**7.5 (3.7-15)**
	**2.5 (2.0-3.0)**	**1.9 (1.9-2.0)**	**4.3 (3.7-5.0)**		**6.7 (2.9-15)**
Tiredness/problem with concentration	**3.0 (2.5-3.6)**	**1.7 (1.6-1.8)**	**2.5 (2.3-2.6)**	**2.0 (1.5-2.7)**	**2,3 (1.2-4.6)**
	0.6 (0.4-1.0)	**4.2 (3.9-4.4)**	**2.5 (2.3-2.6)**	**3.0 (2.1-4.4)**	**4.9 (2.6-8.9)**
Visited physician	**1.4 (1.1-1.9)**	**1.6 (1.5-1.6)**	**2.3 (2.2-2.4)**	**1.2 (1.0-1.6) 1.4 (1.0-1.9)**	1.0 (0.5-1.9)
	**1.9 (1.4-2.6)**	0.9 (0.9-0.9)	**4.4 (4.2-4.7)**		1.5 (0.9-2.5)

^1^ [135]; ^2^ [59]; ^3^ [18]; ^4^ [60],[69],[70],[71]; ^5^ [136]

Summarising results from five studies (Table 1) could easily be fitted into the model (Figure 2). In columns one and two the results put forward that violence both during a child´s development, i.e., childhood/adolescence, and in the present situation (dependency on a violent home environment and/or exposure to violence during adolescence or young adulthood), may be connected to ill health through stress. Results in columns one to five taken together also potentially make it potentially feasible that accumulating exposure to violence and wear and tear on the body evolve into ill health. In study 1-3 and 5 the OR were adjusted for economic margin, educational level, daily smoking, employment status, and civil or marital status. In the childhood/adolescent studies the socio-demographic and socio-economical levels concerned the families. The adult and elderly studies were adjusted for age. The reason for choosing the studies summarised in table 1 were 1; same sample frame, 2; similar sampling technique, and 3; adequately similar time periods. Post estimation was used. In several studies [28],[71],[117],[137] adverse family environment, current violence, and possibly an important transition from adolescence into young adulthood all play a role in the model of understanding exposure to violence and adult ill health.

## 5. Concluding remark

There is a still growing interest and increasing evidences for long-term biological, social, psychological processes that affect adult health [76],[95],[99],[104]. Some of these explanations often have a broad focus on the whole childhood when for instance the acquisition of personal capital [138],[139] is rapid and on late adolescence and young adulthood when many key transitions are made [10]. These processes may run in parallel and interact [75]. Childhood adversity, e.g., may physiologically alter physical growth [140] and socially set the individual on a life trajectory that includes increased risk of exposure, during adulthood, to violence [141] and ill-health [142]. The former is a critical period effect, the latter represents risk accumulation; and these can interact to influence future health.

Trying to fit together all parts and connections of the life course into a model would be tremendously difficult. However, present a model with clear, examinable, and testable different main parts and interconnections that could be fitted/re-fitted into a new model. This new model then could be potentially confirmed or refused and supported with references. There is strength in simplicity when trying to model a phenomenon and even more a strength when the model is sufficiently fitted using exposure to self-reported violence becoming manifest in adverse health outcomes. Theory should be a purely deductive structure from a small number of rather general hypotheses and links to or analogies with already established laws maintained [143].

## 6. Future directions

To challenge the confines of knowledge in the research on violence exposure and ill health; prospective population-based studies should be the preferred method for doing research.

One of the areas that need to be more thoroughly understood is the long-term mechanisms involved in violence exposure and ill health. An understanding of a potentially explanatory life course model would perhaps reveal the internal relationship between violence exposure and ill health.

In, sum, the model suggests that those who experienced severe earlier life events in the form of self-reported exposure to violence are at greater risk of future ill-health compared to those not experienced and self-reported exposure of violence. Three possible backgrounds or present prerequisites combined with two possible pathways resulting in future ill-health. Although evidence supports different aspects of the model, more research is clearly needed.

One of the model's primary premises is that early adversity (violence) leads to greater stress sensitivity, which in turn puts people at greater risk for physical or psychological dysfunction or dysregulation. There are at least two ways to test this part of the model. One way would be to measure acute stress in relation to violence, and evaluate differential stress-induced physiological or psychological changes. Promising results using hair as a retrospective biomarker of increased cortisol production have been presented. These results might implicate that the dysregulation of the hypothalamic-pituitary-adrenal (HPA) axis may be more subtly involved in development and/or maintenance of psychopathology [144],[145],[146]. The other way would be to evaluate those with elevated stress levels and their physical or psychological responses when confronted with violence. This would be particularly important to understand if the model applies to more chronic stressors. Prospective studies repeating the findings of an association between violence exposure and ill health would strengthen the claim of a potential causal relationship between exposure to violence and ill health. Being able to show a possible relationship between violence exposure and chronic conditions such as diabetes, high blood pressure, or cancer would be welcome and further forward our knowledge.

In trying to get the full picture, however, a life course perspective is necessary. From the cradle to the grave is in many ways an accurate expression of the point of attack that public health research needs to have if it is going to disentangle the connection between violence and deteriorated health through the life course.

As research constantly increases our knowledge, reality does not always convert newly gained insights into societal changes. Several policy initiatives for child protection have been introduced since the 1970s. Even so, researchers in Australia, Canada, New Zealand, Sweden, the UK, and the US have not recorded any consistent evidence for a decrease in indicators of child maltreatment. They have noted falling rates of violent death in a few age and country groups, but these decreases only coincided with reductions in admissions to hospital for maltreatment-related injury in Sweden and Canada [147].

Public health workers should never give up, researching and struggling through policies or the handling of other societal changes.
